# An integrated overview of the bacterial flora composition of *Hyalomma anatolicum*, the main vector of CCHF

**DOI:** 10.1371/journal.pntd.0009480

**Published:** 2021-06-09

**Authors:** Nayyereh Choubdar, Fateh Karimian, Mona Koosha, Mohammad Ali Oshaghi

**Affiliations:** Department of Medical Entomology and Vector Control, School of Public Health, Tehran University of Medical Sciences, Tehran, Iran; The University of Texas Medical Branch at Galveston, UNITED STATES

## Abstract

The microbial flora associated with *Hyalomma anatolicum* ticks was investigated using culture-dependent (CD) and independent (next generation sequencing, NGS) methods. The bacterial profiles of different organs, development stages, sexes, and of host cattle skins were analyzed using the CD method. The egg and female gut microbiota were investigated using NGS. Fourteen distinct bacterial strains were identified using the CD method, of which *Bacillus subtilis* predominated in eggs, larval guts and in adult female and male guts, suggesting probable transovarial transmission. *Bacillus velezensis* and *B*. *subtilis* were identified in cattle skin and tick samples, suggesting that skin is the origin of tick bacteria. *H*.*anatolicum* males harbour lower bacterial diversity and composition than females. The NGS analysis revealed five different bacterial phyla across all samples, Proteobacteria contributing to >95% of the bacteria. In all, 56611sequences were generated representing 6,023 OTUs per female gut and 421 OTUs per egg. Francisellaceae family and *Francisella* make up the vast majority of the OTUs. Our findings are consistent with interference between *Francisella* and *Rickettsia*. The CD method identified bacteria, such *B*. *subtilis* that are candidates for vector control intervention approaches such paratransgenesis whereas NGS revealed high *Francisella* spp. prevalence, indicating that integrated methods are more accurate to characterize microbial community and diversity.

## Author summary

Crimean-Congo hemorrhagic fever (CCHF) is a viral disease transmitted by hard ticks (Ixodidae: Ixodida) that has a 10–40% fatality. While more than 3 billion people are at risk acquiring the disease, on the order of 10,000–15,000 people are infected and over 500 people die every year from the disease. The main vector and reservoir of the CCHF virus are ticks of the genus *Hyalomma*. As the microbiota influences vector competence, its manipulation may be used for vector control. In this study, we characterize cultivable and non-cultivable bacteria from *H*. *anatolicum* ticks. Field collected samples were processed for aerobic culture and 16s rRNA Next Generation Sequencing (NGS). The cultivable bacterium *Bacillus subtilis* was found in all samples analysed, including *H*. *anatolicum* males, females, larval guts and host skin. NGS revealed *Francisella* spp as the most common non-cultivable bacteria in *H*. *anatolicum* ticks. Bacterial OTUs varied between eggs and female guts, indicating that community structure varies by tick developmental stage. Our results indicate that *B*. *subtilis* is be a potential paratransgenesis candidate for vector control intervention. Integrated methods (bacteria culture, NGS) are recommended for more accurate characterization of microbial community and diversity.

## Introduction

Tick-borne diseases (TBDs) are imposing a growing burden for human and animal health worldwide. Ticks are obligate blood-feeders and can transmit to humans and animals a wide variety of pathogenic agents such as arboviruses, bacteria, and parasites. Hard ticks (Ixodidae) typically have three life stages (larva, nymph, adult) and feed on distinct host species at each developmental stage, making them important sources of zoonotic diseases [1].

*Hyalomma anatolicum* (Acari: Ixodidae) is the most important tick involved in transmission of the Crimean-Congo hemorrhagic fever (CCHF) virus in Iran [2–4]. In addition of CCHF, *H*. *anatolicum* is also a vector of a wide variety of agents such as Thogoto virus, Wad Medani virus (WMV), *Theileria* sp., *Ehrlichia* sp., *Rickettsia* sp., *Babesia ovis* [5–13], causing transitory lameness [14] and *Coxiella burnetii* [15].

Symbiotic and commensal microbes can confer numerous unfavourable, neutral, or beneficial effects to their arthropod hosts, and can play several roles in fitness, development, nutritional adaptation, oviposition, egg hatching, larval survival, reproduction, defence against environmental stress, and immunity [16–24]. Non-pathogenic microbes may also play a role in transmission of tick-borne pathogens (TBP), with many possible consequences for both animal and human health [25].

The hard tick midgut is colonized by symbiotic, environmentally acquired, and maternally transmitted bacteria. Characterization of *H*. *anatolicum* microbiota requires the isolation of the natural bacteria via culture. The cultivable bacteria may be used for vector control interventions such as paratransgenic and RNAi approaches [26,27] that may be explored to render ticks refractory to pathogen infection. On the other hand, non-cultivable bacteria are important components of the tick microbiome. They include endosymbionts beneficial for tick survival such as *Coxiella* spp., [28–30], *Rickettsia* spp., [31–34], *Francisella* spp., [33,35] and “*Candidatus* Midichloria mitochondrii” [36], and pathogenic bacteria such as *Anaplasma*, *Borrelia*, *Ehrlichia*, *Francisella* and *Rickettsia* [37–40]. The introduction of next-generation sequencing (NGS) technologies has permitted the rapid and economic characterization of these microbial communities [37] in contrast to the previously used Sanger sequencing. Recently the number of studies using NGS to investigate the microbial diversity and composition of ticks has expanded [24,37]. There are nine hyper variable regions (V1-V9) of the bacterial 16S ribosomal RNA gene (16S) that can be used to identify bacterial taxa, the V1-V3, V3-V5, V4-V5 regions being the most commonly used. The 454 (Roche) pyrosequencing, Ion Torrent (Thermo Fisher) sequencing by semiconductor ion detection and Illumina MiSeq platforms using fluorescent dye detection have been the most commonly tick microbiome sequencing methods [37,41,42].

Recently, several reports of microbial composition associated with different development stages, sex, and organs, especially the digestive tract of ticks have been published [37]. However, none addressed the bacterial composition of *Hyalomma* ticks, the main vector of CCHF virus. Two studies assayed *H*. *asiaicum* RNA virus infection [43,44]. The aim of this study is to report characterization of the microbiome of *H*. *anatolicum* ticks and their host’s skins using culture-dependent and NGS approaches to identify potential bacterial candidate/s for use with paratransgenesis or RNAi approaches.

## Methods

### Ethics statement

This study followed the guidelines of the institutional ethical committee (Tehran University of Medical Sciences, TUMS). The protocols were approved by TUMS ethical committee under registry IR.TUMS.SPH.REC.1395.926.

### Tick collection and identification

**Fig 1:**
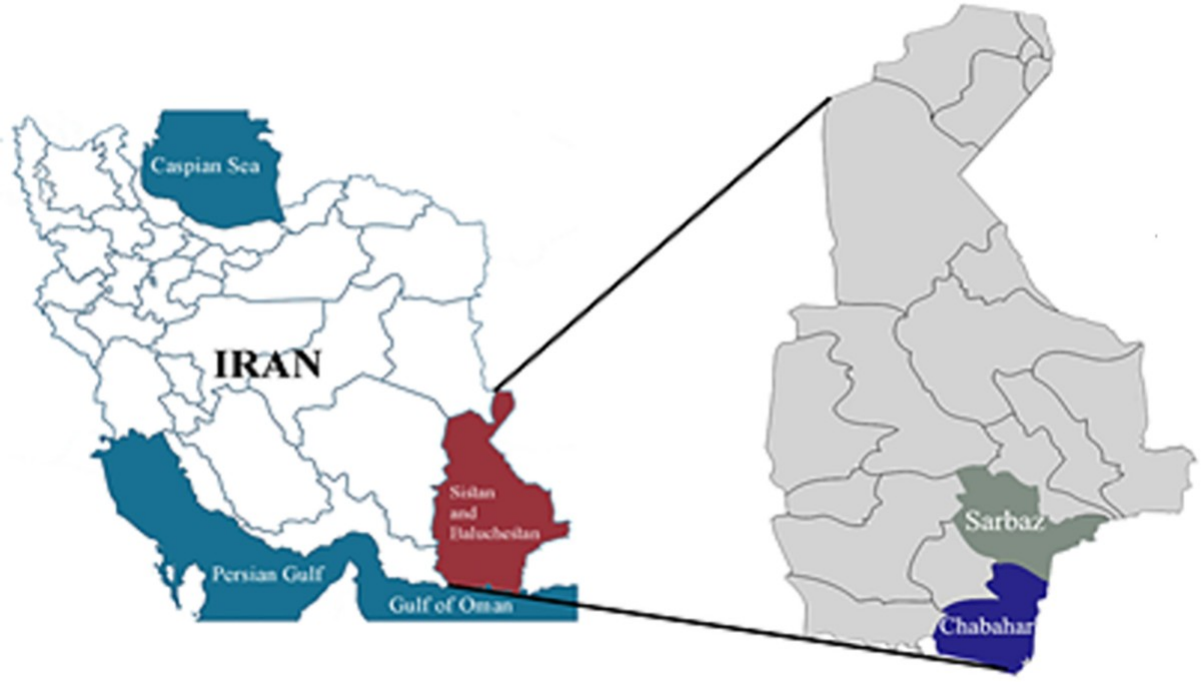
Map of Iran showing the locations in which tick samples were collected (https://commons.wikimedia.org/wiki/File:Map_of_Iran.png).

This study was carried out in two closely Crimean-Congo Hemorrhagic Fever (CCHF)-endemic districts (Sarbaz and Chabahar) located in south-east corner (*Sistan and Baluchestan* Province) of Iran (Fig 1). Sistan and Baluchestan is one of the largest provinces of Iran (181,785 km^2^) that borders Pakistan and Afghanistan and has subtropical climate. *Hyalomma* ticks were collected from cattle from this region and kept alive in separate sterile 50 ml Falcon tubes containing a piece of filter paper until their dissection. They were transferred to the laboratory of insect molecular biology, School of Public Health, Tehran University of Medical Sciences (SPH-TUMS). Ticks were identified morphologically to the species level using taxonomic keys [45,46]. About 20% (n = 70) female ticks were selected randomly for NGS analysis. Subsets of engorged females (n = 120) were allowed to lay eggs, and 170 eggs were used for either CD or NGS bacterial analysis (Table 1).

**Table 1:** Details of the *H*. *anatolicum* specimens collected for microbiome analysis.

Location	Latitude & longitude	Development stage & Organ (n)	Method & No of specimens tested (n)
Sarbaz	26°37’35.5”**N** 61°15’42.9”**E**	Egg batch (85)	CD (50) NGS (35)
		Larva gut (127)	CD (127)
		Male gut / MT (59/59)	CD (59/59)
		Female gut / MT (164/164)	CD (129/129) NGS (35)
Chabahar	26°14’27.5”**N** 61°24’10.0”**E**	Egg batch (35)	CD (50) NGS (35)
		Male gut / MT (87/87)	CD (87/87)
		Female gut/MT (193/193)	CD (158/158) NGS (35)

### Specimen processing and isolation of midgut

A total 630 *H*. *anatolicum* ticks, including adults (n = 503) and larvae (n = 127), were identified and used for bacterial isolation (Table 1). Ticks were individually surface sterilized as described by Portillo et al [47]. Briefly, tick was immersed for 5 min in 70% ethanol and then rinsed with autoclaved double distilled water. Each specimen was then fixed in sterilized paraffin by their legs and UV sterilized under sterile conditions in a Class II biosafety cabinet. Lateral cuts were made with a sterile scalpel and dorsal integument was removed. To understand bacterial diversity, guts and Malpighian tubules (MT) were teased away from other organs using ultra-fine sterile forceps. Between dissections, forceps and scalpel blades were sterilized with bleach (10%) and flame. To reduce laboratory-derived contamination; we used workstations, sterile gloves, pipette tips with filters, and PCR grade RNAse-free water and the experiments performed under laminar flow hoods. Guts and MTs were transferred separately to 100 μL sterile phosphate-buffered saline (PBS) (pH 7.2), and homogenized. Swabbing of the cattle’s ear (the preferred site for tick attachment) was performed after washing with distilled autoclaved water to remove sediment, dirt, and transient bacteria. Swabbing consisted of five strokes along each ear. Swabs were placed directly in Falcon tubes containing brain heart infusion (BHI) broth medium. Guts and MTs were pooled separately according to the sex and development stages, up to 5 individuals per pool. A total of 560 guts or MTs from field-collected *H*. *anatolicum* ticks were used for culture-dependent identification and 70 female guts representative of different locations and hosts were analysed by NGS.

A subset of the engorged live female ticks was maintained in the insectarium until oviposition. Engorged females were kept in glass vials at about 70–80% relative humidity and 27–28°C under a photoperiod of 14:10 hours (light: dark) until oviposition. Pools of up to five egg batches were initially washed with distilled water followed by three 70% ethanol washes and then rinsed with distilled water, air-dried and homogenized with glass pestles in 1ml of sterile PBS. The egg homogenates were plated for bacteria. Seventy eggs were also processed for NGS identification. For NGS analysis, due to shortage of funds, we processed only pool female gut and egg samples (Table 1). The final water used for rinsing the cuticles and egg batches were used as negative controls and plated in parallel. To assess the environmental contamination, the cuticles, as an environmental control, was removed from the tick carcass and were subjected to DNA extraction by phenol chloroform method and PCR amplification of 16s rRNA gene. Where the negative control was positive the specimen was eliminated from further analysis. Frequent changes of gloves were used to avoid RNAse-DNAse contamination. Surface sterilization of workstation by bleach (10%) followed by alcohol (70%) was performed prior and after each experiment. Also we have used autoclaved instruments before and after handling each sample, avoid talking, sneezing, and coughing, and touching the area where DNA may exist.

### Isolation of bacteria

#### The culture-dependent method

A 1 ml of each homogenized pool sample was added to Falcon tubes containing 5 ml of brain heart infusion (BHI) broth and incubated overnight at 37°C and 100 rpm. To obtain single colonies, 100 μl of the overnight cultures were spread onto LB agar plates and incubated at 37°C for 18–24 h. DNA was extracted from individual colonies using either a boiling method (STET buffer) and/or routine phenol/chloroform. Phenol/chloroform DNA extraction method was used for the isolates with hard cell walls that had not yielded proper DNA by the boiling method [48].

The 16S rRNA gene was amplified using forward primer 16suF 5′-GAGTTTGATCCTGGCTCAG-3′ and reverse primer 16suR 5′-GTTACCTTGTTACGACTT-3′ as reported by Weisburg [49] yielding a 1,500 bp fragment. The PCR amplification was performed using Maxime PCR PreMix Kit (i-Taq) in 20 μl reaction mixtures containing 1 μl of 10 μM both forward and reverse primers and 1–2 μl (~0.1 μg) of extracted genomic DNA. Three no-template controls including PCR grade RNAse-free water, the water used for washing specimens following ethanol sterilization, and the sterilized cuticles were used to detect any bacterial and/or DNA contamination in the culture media and amplification reagents.

The PCR reactions were performed under the following conditions: initial denaturation at 94°C for 1 min, followed by 35 cycles of 95°C for 30S, annealing at 57.5°C for 40 s, 72°C for 30 s and a final extension at 72°C for 8 min. The PCR product were fractionated on a 1% agarose gel and visualized using an UV transilluminator. PCR Purification Kit (Qiagen, Germany) was used for the purification of PCR products before sequencing.

All successfully amplified 16S rRNA amplicons were directionally sequenced using the same amplification primers obtained from Bioneer Company (S. Korea). The sequences were analysed using five databases: EzTaxon-e [http://eztaxon-e.ezbiocloud.net], NCBI (16S rRNA sequences) [http://blast.ncbi.nlm.nih.gov/Blast.cgi], NCBI (Nucleotide collection) [http://blast.ncbi.nlm.nih.gov/Blast.cgi], leBIBI [http://umr5558-sud-str1.univ-lyon1.fr/lebibi/lebibi.cgi], and Blast2Tree [http://bioinfo.unice.fr/blast]. Sequence homology analysis was based on the number and quality of nucleotides in a given sequence and setting defaults of the databases such as cultivable and or non-cultivable, type and non-type specimens. In case of discrepancies among different databases, species identifications were based on either the most common nomenclature among the results of the four databases or on the basis of the highest percentage similarity. Sequences have been submitted to GenBank under Accession Numbers MN399911, MN399915-MN399917, MN399925-MN399926, MN399929-MN399930, MN399941, MN399950-MN399951 and MT355659-MT355661.

#### The culture-independent method

DNA was extracted from homogenized gut or egg pools using DNA extraction kit (QiAamp DNA micro kit) following the manufacturer’s recommended protocol. DNA was stored at -20°C until used for sequencing.

The 16S rRNA gene hyper variable V3 region PCR amplified using fusion degenerate primers 341F (5’-CCTACGGGAGGCAGCAG -3’) and 518R (5’- ATTACCGCGGCTGCTGG -3’) and was sequenced on an Illumina Miseq platform. The amplified fragment was approximately 342 bp and raw data were screened and assembled by QIIME. The UCLUST method was used to cluster the sequences into Operational Taxonomic Units (OTUs) at an identity threshold of 97%. Each library pool was sequenced on a Junior+ System Genome Sequencer.

### Data analysis

Cytoscape Software (http://www.cytoscape.org) was used to visualize bacterial richness and egg and female gut shared bacteria [50]. GraphPad Prism software v.5.00 for Windows (GraphPad, San Diego, USA) was used for graphical representation.

## Results

### Bacteria composition using a culture-dependent approach

Using a culture-dependent method, a total of 97 bacterial strains were identified from different developmental stages, organs and sexes of the field-collected *H*. *anatolicum* and from the skin of their host (S1 Table). Bacteria were plated on BHI agar and identified based on 16S rRNA sequencing. Eleven bacterial strains were recovered from the *H*. *anatolicum* guts and three strains from cattle’s skin (Table 2). Except for one Acintobacteria, all strains belong to the Firmicutes phylum. All cultivable bacteria were Gram positive (G+). A majority (10 out of 14, 71.4%) of the G^**+**^ strains from *H*. *anatolicum* guts and animal skins belong to the *Bacillus* genus. G^**+**^ *Paraclustridium*, *Enterococcus*, and *Micrococcus* were also recovered. In *H*.*anatolicum* guts, the species found were *Bacillus subtilis*, *B*. *licheniformis*, *B*. *velezensis*, *B*. *oceanisedimini*, *Micrococcus aoeverae*, *Enterococcus lactis*, and *Paraclustridium benzoelyticum*. Only two strains of *P*. *benzoelyticum* and *B*. *licheniformis* were recovered from eggs, the later one also was recovered from female guts. *Micrococcus aoeverae* was shared between the guts and MTs of ticks. Of the six bacterial species identified in adults, only *B*. *subtilis* was shared between the two sexes (Table 2). *B*. *subtilis* has also been found in larval guts and in host skin (Table 2). In addition to *B*. *subtilis* the microbiome of cattle’s skin included *B*. *velezensis* which is also found in tick guts.

**Table 2:** Bacterial profile of *H*. *anatolicum* ticks and their host skin (ear) revealed by culture dependent method.

No	Bacteria Species	Development Stage	Sex	Organ or origin	Location	Gen Bank ID number
**1**	*Enterococcus lactis*	Adult	Male	Gut	Chabahar	MN399911
**2**	*Bacillus subtilis*	Adult	Female	Gut	Sarbaz	MN399915
**3**	*Bacillus subtilis*	Adult	Male	Gut	Sarbaz	MN399916
**4**	*Bacillus velezensis*	Adult	Male	Gut	Sarbaz	MN399925
**5**	*Bacillus oceanisedimini*	Adult	Female	Gut	Sarbaz	MN399926
**6**	*Bacillus licheniformis*	Adult	Female	Gut	Chabahar	MN399929
**7**	*Micrococcus aoeverae*	Adult	Female	Gut	Chabahar	MN399950
**8**	*Micrococcus aoeverae*	Adult	Female	MT	Chabahar	MN399951
**9**	*Bacillus subtilis*	Larvae	NA	Gut	Sarbaz	MN399917
**10**	*Bacillus licheniformis*	Egg	NA	Egg	Chabahar	MN399930
**11**	*Paraclustridium benzoelyticum*	Egg	NA	Egg	Sarbaz	MN399941
**12**	*Bacillus subtilis*	NA	NA	Cattle skin	Chabahar	MT355661
**13**	*Bacillus subtilis*	NA	NA	Cattle skin	Sarbaz	MT355660
**14**	*Bacillus velezensis*	NA	NA	Cattle skin	Sarbaz	MT355659

**Fig 2:**
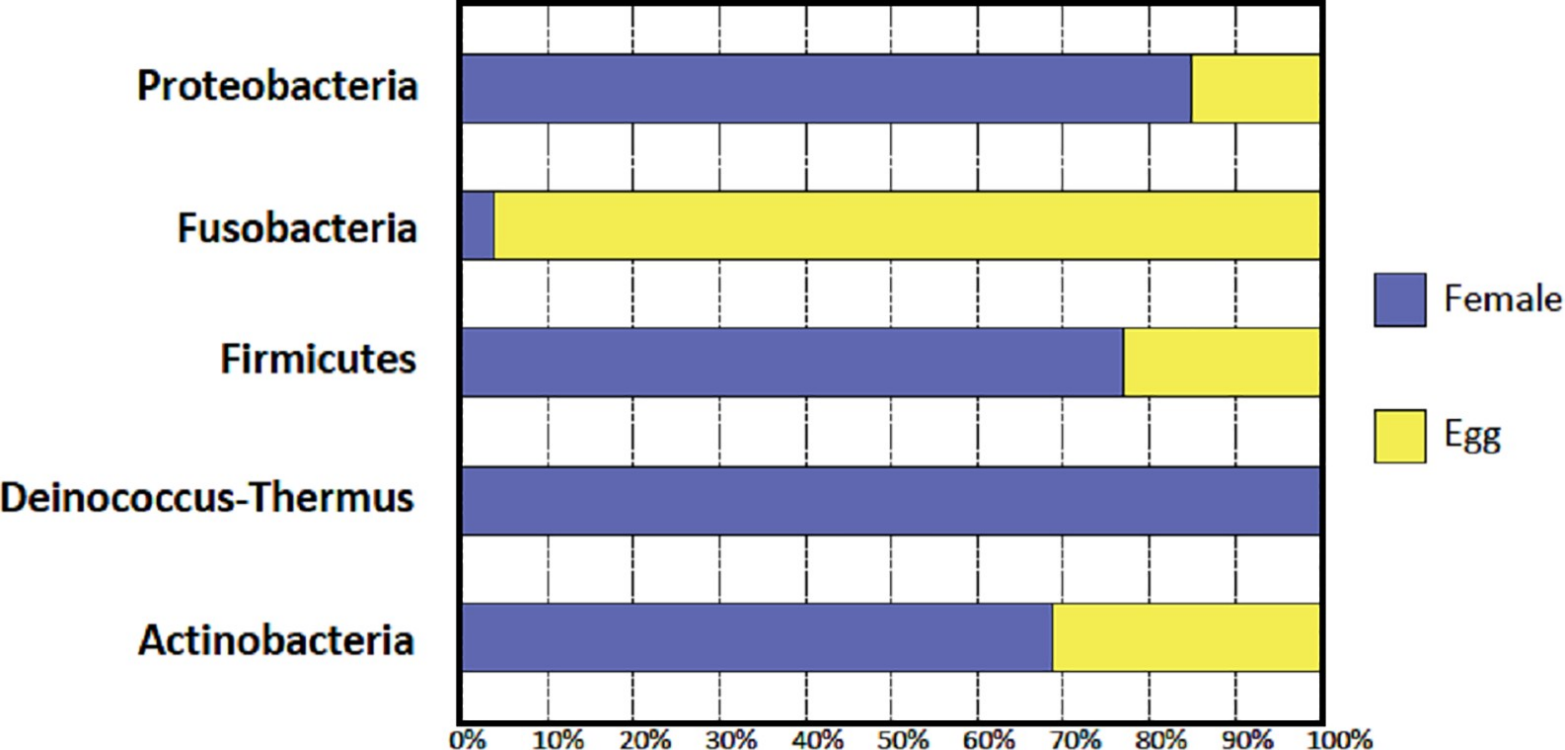
Relative abundance of *H*. *anatolicum* gut and egg bacterial phyla revealed by 16S rRNA gene sequencing on the Illumina MiSeq platform.

**Fig 3:**
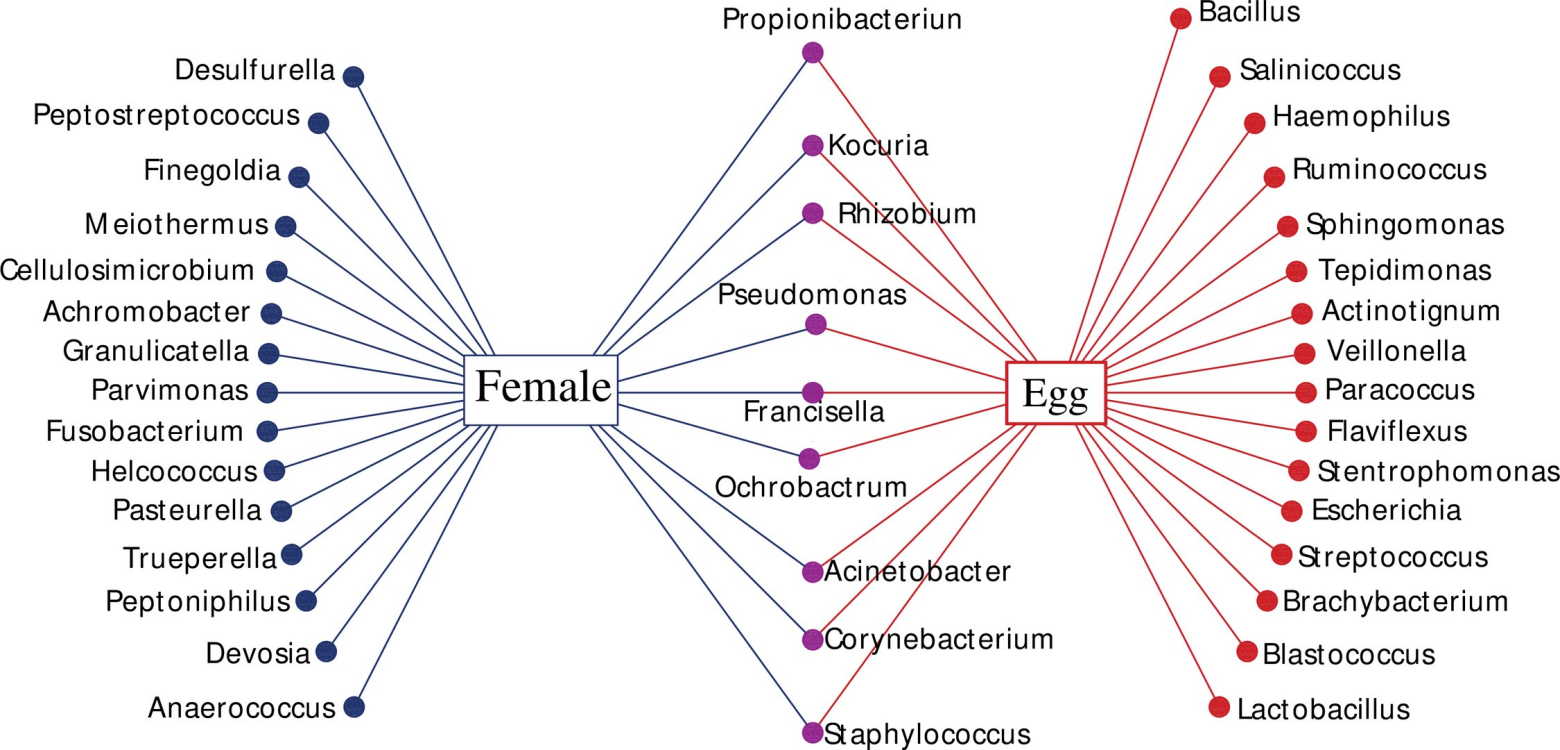
Network analysis showing the shared and non-shared *H*. *anatolicum* female gut and egg bacteria genera revealed by NGS.

The NGS method was used to characterize the microbiome of field collected *H*. *anatolicum* female guts and eggs. A 346 bp fragment of the hypervariable V3 region of the 16S rRNA gene was PCR amplified from the genomic DNA pools (female gut and egg) using specific universal primers and sequenced using the Illumina-MiSeq platform. A total of 56,611 sequences were generated that were classified into 6,023 operational taxonomic units (OTUs) per female gut and 421 OTUs per egg. The following phyla were identified: Proteobacteria, Actinobacteria, Firmicutes, Deinococcus-Thermus and Fusobacteria. The relative abundance of different female and egg bacterial phyla is summarized in Fig 2. The phylum Proteobacteria makes up nearly all the RPA (relative present abundance) and contributed to 94.9%, and 96.1% of the bacterial sequences in eggs and female guts, respectively. These bacteria belonged to 32 families and 39 genera. A total of 24 and 25 genera were found in female guts and egg samples, respectively (Table 3). Next generation sequencing revealed that *Francisella* makes up the vast majority of the RPA, making up 96.8 and 92.1% of the female gut and egg bacterial community, respectively. The following nine (out of 40) bacterial genera *Kocuria*, *Propionibacterium*, *Corynebacterium*, *Staphylococcus*, *Ochrobactrum*, *Acinetobacter*, *Rhizobium*, *Pseudomonas* and *Francisella*, were shared between the egg and female gut samples (Table 3, Fig 3).

**Table 3:** Taxonomic, characters, and the number of operational taxonomic units (OTUs) in *H*. *anatolicum* eggs and female guts revealed by NGS.

Phylum	Family	Genus	Gram + / -	Characters	No. of OTUs in eggs	No. of OTUs in female guts
Actinobacteria	Actinomycetaceae	*Actinotignum*	**+**	Pathogen	5	0
		*Flaviflexus*	**+**	Pathogen	6	0
		*Trueperella*	**+**	Pathogen (In cattle)	0	140
	Promicromonosporaceae	*Cellulosimicrobium*	**+**	Pathogen	0	10
		*Propionibacterium*	**+**	Non-pathogen (opportunistically)	23	9
	Corynebacteriaceae	*Corynebacterium*	**+**	Non-pathogen (opportunistically)	67	82
	Geodermatophilaceae	*Blastococcus*	**+**	Enviromental	9	0
	Dermabacteraceae	*Brachybacterium*	**+**	Enviromental	17	0
	Micrococcaceae	*Kocuria*	**+**	Non-pathogen	15	18
Firmicutes	Bacillaceae	*Bacillus*	**+**	Pathogen & Non-pathogen	3	0
	Staphylococcaceae	*Salinicoccus*	**+**	Non-pathogen	17	0
		*Staphylococcus*	**+**	Pathogen	49	84
		*Streptococcus*	**+**	Non-pathogen	58	0
	Peptoniphilaceae	*Finegoldia*	**+**	Pathogen (Opportunistically)	0	58
		*Parvimonas*	**+**	Pathogen	0	130
		*Peptoniphilus*	**+**	Non-pathogen	0	15
	Peptostreptococcaceae	*Peptostreptococcus*	**+**	Non-pathogen	0	63
	Ruminococcaceae	*Ruminococcus*	**+**	Pathogen	8	0
	Veillonellaceae	*Veillonella*	-	Non-pathogen	10	0
	Heliobacteriaceae	*Helcococcus*	**+**	Pathogen	0	195
	Clostridiales	*Anaerococcus*	**+**	Pathogen	0	5
	Carnobacteriaceae	*Granulicatella*	**+**	Pathogen	0	9
	Lactobacillaceae	*Lactobacillus*	**+**	Non-pathogen	35	0
Proteobacteria	Brucellaceae	*Ochrobactrum*	-	Non-pathogen	49	61
	Hyphomicrobiaceae	*Devosia*	-	Enviromental	0	10
	Rhizobiaceae	*Rhizobium*	-	Enviromental	14	5
	Rhodobacteraceae	*Paracoccus*	-	Enviromental	56	0
	Sphingomonadaceae	*Sphingomonas*	-	Pathogen (Nosocomial infections)	3	0
	Alcaligenaceae	*Achromobacter*	-	Enviromental	0	14
	Comamonadaceae	*Tepidimonas*	-	Non-pathogen	16	0
	Desulfurellaceae	*Desulfurella*	-	Non-pathogen	0	7
	Enterobacteriaceae	*Escherichia*	-	Pathogen	2	0
	Yersiniaceae	*Haemophilus*	-	Non-pathogen	15	0
		*Pasteurella*	-	Pathogen	0	178
	Moraxellaceae	*Acinetobacter*	-	Pathogen (Nosocomial infections)	25	27
	Pseudomonadaceae	*Pseudomonas*	-	Pathogen (Opportunistically)	80	40
	Francisellaceae	*Francisella*	-	Pathogen	7552	42451
	Xanthomonadaceae	*Stenotrophomonas*	-	Pathogen (Opportunistically)	62	0
Deinococcus- Thermus	Thermoaceae	*Meiothermus*	**+**	Pathogen (In bird)	0	13
Fusobacteria	Fusobacteriaceae	*Fusobacterium*	-	Pathogen	0	213

## Discussion

Results of NGS analysis revealed the presence of endosymbionts such as *Francisella* spp. and *Candidatus*, as well as pathogenic, environmental, and skin-associated bacteria in the gut of *H*. *anatolicum*. *Francisella* spp., important tick-borne pathogens (TBPs) of humans and animals, were the dominant bacteria (more than 92% of OTU reads) associated with *H*. *anatolicum* guts and eggs. *Francisella* and Francisella-like endosymbiotic bacteria (FLEs) are transovarially transmitted and potentially obligate endosymbionts. These bacteria have also been identified in *Ornithodoros moubata* ovaries and Malphigian tubules [51] and in several hard ticks [16,33,35,52,53]. Interestingly, NGS analysis revealed that none of the *H*. *analoticum* ticks harboured other known TBPs such as *Ehrlichia*, *Anaplasma*, *Babesia*, and spotted fever group *Rickettsia* (SFGR). It is known that *Rickettsia* and *Francisella* were negatively correlated in the ticks [54] and that *Francisella* outcompetes other bacterial genera [33]. However, it is noticeable that previous studies have shown the presence of *Ehrlichia*, *Anaplasma*, and *Babesia* in the ticks of our study region [55–57], therefore further studies are needed to confirm the hypothesis that FLEs interfere with the ability of *Hyalomma* ticks to be infected with *Ehrlichia*, *Anaplasma*, *Babesia* and SFGR.

In this study different microbial communities were found between the *H*. *anatolicum* gut and MT and the guts and/or MT with eggs studied. This is in agreement with previous studies indicating microbial variation among anatomical regions within the tick such as the reproductive tract, midgut, and the salivary glands [28,34,57–60]. NGS analysis revealed considerable differences in the frequency of bacteria in female guts and eggs (6,023 versus 421 OTUs). However, the diversity between the bacterial community of the guts and eggs was not significant (24 versus 25 with 9 shared genera). Culture dependent method revealed a great variation in frequency and diversity of bacteria among gut, egg, and Malpighian tubule (8 versus 2 versus 1). *Micrococcus aoeverae* was the only Malpighian tubule bacterium also found in guts, suggesting it is exceptional in its capacity to migrate from midgut to Malpighian tubules, and colonize in this organ. In addition, some bacteria were shared between eggs and guts, indicating possible transovarial transmission from females to eggs and presumably to the next generation.

It seems that the location have effect on the results of tick bacterial community where no tick associated with *B*. *subtilis* in Chabahar district, despite this bacterium being found in the skin of cattle in the region. Further field studies are required to verify these preliminary findings. On the other hand, although all of the controls which were used in this study were not environmentally contaminated, the use of 70% ethanol for 5 min, as the only method used in this study, may not be effective enough especially for *B*. *subtilis* spores.

*Bacillus licheniformis*, found in *H*. *anatolicum* eggs, produces microbial polysaccharides with multiple bioactivity including antibiofilm activity against Gram-negative (*Pseudomonas aeruginosa* and *Proteus vulgaris*) bacteria, *Candida albicans*, and mosquito larvae [61]. This may partially explain presence of only gram positive bacteria in the culture media of our study. Microbial polysaccharide insect toxicity may play a role in protection of tick eggs against insect predators. These observations deserve further consideration for entomological applications of this bacterium species.

Among environmental and host-related factors that may influence diversity and composition of the *H*. *anatolicum* microbiome, we have assessed the effect of sex, organs, and developmental stages. The CD method showed that *H*. *anatolicum* male guts harbour lower microbiome diversity and composition than that of females (2 versus 6). Only *B*. *subtilis* was shared between the two sexes. It has been reported that females have higher [30,62] or lower [34,54,63] relative bacterial abundance than of males, while other researchers indicated that males and females adults differed only in their community structure, for example, males containing more *Rickettsia* and females containing more *Coxiella* [64]. These data suggest that tick microbial community is dynamic.

Our NGS analysis identified pathogenic bacteria associated with *H*. *anatolicum* ticks. However, these ticks may harbour additional yet-undiscovered human or animal pathogens and pathogenicity of such bacteria remains to be determined. The fact that *H*. *anatolicum* is among the most frequent ticks that come in contact with humans and cattle in Iran [4,65] emphasizes the need to characterize all *H*. *anatolicum*-associated microbes to determine the full spectrum of agents that can be transmitted by this tick.

There is current interest in the use of microorganisms as biological control agents of vector borne diseases [66]. Strategies could be developed to manipulate the certain components of the tick microbiome to decrease the vectorial capacity of ticks by hindering pathogen acquisition, development, and horizontal and vertical transmission. Similar microbial management strategies could be developed for ticks which promote the growth of endosymbiotic bacteria to reduce the acquisition of pathogens. Here, we have isolated a strain of the non-pathogenic *Bacillus* species (*B*. *subtilis*) from *H*. *anatolicum*, which was previously introduced as a promising candidate for paratransgenic approach [67,68]. We have identified *B*. *subtilis* in *H*. *anatolicum* eggs, female midguts, males, larvae, as well as cattle’s skin. Moreover, it was shown that *H*. *asiaticum* ticks can acquire bacteria from host skin while blood feeding [27]. *B*. *subtilis* has been isolated from different arthropods including ticks [69,70], and has potential to be used for control of TBDs. In addition to being non-pathogenic, it is easily cultured and genetically manipulated [67]. The use of symbiotic bacteria expressing dsRNA in a paratransgenic approach is a new method for the control of vector-borne disease [71,72] and has already been used for reducing tick pathogens [73–76]. For using *B*. *subtilis* for paratransgenic approaches, it will be important to examine its capacity to efficiently colonize the gut, reproductive organs, or salivary glands of *Hyalomma* spp., and to express enough effector molecules or dsRNA to inhibit the target gene.

## Conclusions

The culture-dependent approach revealed a bacterial community diversity comprising gram positive bacteria belonging to mostly Firmicutes phyla, among which *B*. *subtilis* was the dominant bacterium. *Bacillus licheniformis* was isolated from eggs and female guts suggesting possible transovarial transmission as well as protective role against insect predators. However, other tick tissues, especially ovaries, should be analysed to support this premise. Presence of *B*. *subtilis* in the guts of females, males, and larvae of *H*. *anatolicum* ticks as well as their host’s skin suggests that this bacterial species is a potential candidate for paratransgenic and RNAi approaches for prevention of TBPs transmission. High frequency of *Francisella* and lack of *Rickettsia* genus is in agreement with that microbe-microbe interactions phenomena and their influence on microbiome composition and interfere with TBP transmission. Finally integration of culture-dependent and culture–independent method provides better understanding and more extensive and accurate results in terms of the microbial community of vector microbiome.

## Supporting information

S1 Table Details of the bacteria isolated from *H*. *anatolicum* ticks and their host skin revealed by culture dependent method followed by 16sRNA gene sequencing. (DOCX)
