# Supplementary material for: An integrated overview of the bacterial flora composition of Hyalomma anatolicum, the main vector of CCHF
Source: PLoS Negl Trop Dis. 2021 Jun 9;15(6):e0009480. doi: 10.1371/journal.pntd.0009480 (PMC8216544; doi:10.1371/journal.pntd.0009480)
Supplement: S1 Table — (DOCX) [file pntd.0009480.s001.docx]

**Supplementary data**

**S1 Table.** Details of the bacteria isolated from *H. anatolicum* ticks and their host skin revealed by culture dependent method followed by 16sRNA gene sequencing.

| **Location** | **Origin** | **Tick Development**  **stage** | | **No of Specimen tested** | **Organ** | **Bacteria species** | **No of isolates sequenced** |
| --- | --- | --- | --- | --- | --- | --- | --- |
| Chabahar | *Hy. anatolicum* | Adult | Female | 158 | gut | *Micrococcus aoeverae* | 10 |
|  |  |  |  |  |  | *Bacillus licheniformis* | 11 |
|  |  |  |  | 158 | MT | *Micrococcus aoeverae* | 4 |
|  |  |  | Male | 87 | gut | *Enterococcus lactis* | 11 |
|  |  |  |  | 87 | MT | *---* | --- |
|  |  | Egg | | 50 | N/A | *Bacillus licheniformis* | 4 |
|  | Cattle (tick host) | N/A | | N/A | Ear skin | *Bacillus subtilis* | 2 |
| Sarbaz | *Hy. anatolicum* | Adult | Female | 164 | gut | *Bacillus subtilis* | 13 |
|  |  |  |  |  | gut | *Bacillus oceanisedimini* | 7 |
|  |  |  |  | 164 | MT | *---* | --- |
|  |  |  | Male | 59 | gut | *Bacillus velezensis* | 8 |
|  |  |  |  |  |  | *Bacillus subtili* | 4 |
|  |  |  |  | 59 | MT | *---* | --- |
|  |  | Larvae | | 127 | gut | *Bacillus subtilis* | 7 |
|  |  | Egg | | 50 | N/A | *Paraclustridium benzoelyticum* | 3 |
|  | Cattle (tick host) | N/A | | N/A | Ear skin | *Bacillus velezensis* | 10 |
|  |  |  |  |  |  | *Bacillus subtilis* | 3 |
